# Long non-coding RNA PVT1 encapsulated in bone marrow mesenchymal stem cell-derived exosomes promotes osteosarcoma growth and metastasis by stabilizing ERG and sponging miR-183-5p

**DOI:** 10.18632/aging.102406

**Published:** 2019-11-07

**Authors:** Wei Zhao, Pan Qin, Da Zhang, Xichun Cui, Jing Gao, Zhenzhu Yu, Yuting Chai, Jiaxiang Wang, Juan Li

**Affiliations:** 1 Department of Pediatric Surgery, The First Affiliated Hospital of Zhengzhou University, Zhengzhou 450052, China; 2 Department of Respiratory Medicine, The First Affiliated Hospital of Zhengzhou University, Zhengzhou 450052, China

**Keywords:** exosome, lncRNA PVT1, ubiquitination, osteosarcoma, bone marrow mesenchymal cells

## Abstract

Exosomes secreted by bone marrow mesenchymal stem cells (BMSCs) promote osteosarcoma cell proliferation and migration, while the underlying mechanism remains unknown. Since the long non-coding RNA PVT1 has been reported to be upregulated in osteosarcoma cells and contributes to its growth and metastasis, we aim to investigate whether BMSC-derived exosomes promote osteosarcoma growth and metastasis via transporting PVT1 into osteosarcoma cells. The PVT1 expression in BMSC-derived exosomes was markedly higher than that in osteosarcoma cell-derived exosomes. The co-culturing of BMSC-derived exosomes and osteosarcoma cells (Saos-2, MG-63, and MNNG/HOS cell lines) significantly raised PVT1 expression of osteosarcoma cells. The direct binding between PVT1 and the oncogenic protein ERG was confirmed using RNA immunoprecipitation and RNA pull-down assays, and the transported PVT1 promotes osteosarcoma cell proliferation and migration via inhibiting degradation and ubiquitination of ERG. PVT1 also increased ERG expression through sponging miR-183-5p. In summary, our findings indicated that BMSC-derived exosomes encapsulate PVTl and transport it into osteosarcoma cells, and the transported PVT1 promotes tumor growth and metastasis by inhibiting ubiquitination and promoting expression of ERG in osteosarcoma cells. These data provide a novel insight into the mechanism of BMSC-derived exosomes in affecting osteosarcoma progression.

## INTRODUCTION

Osteosarcoma is a malignant bone tumor with the highest morbidity in children and adolescents, and is clinically localized in the metaphysis of long bones [1]. The early metastasis and lung metastasis are commonly seen in osteosarcoma, causing the main mortality of osteosarcoma patients [2]. With the limited treatment of osteosarcoma, it desires better solution to deal with osteosarcoma. In recent years, growing researches have shown that the tumor microenvironment (TME) plays an important role in tumor progression [3, 4]. Bone marrow mesenchymal stem cells (BMSCs) are one of the major components in the TME of osteosarcoma and are corroborated to mediate proliferation and metastasis of tumor cells [5]. More importantly, BMSCs exert their functions partly through secreting active substances, such as exosomes, to activate signal transduction pathways in osteosarcoma cells [6, 7].

Exosomes are nanovesicles of 40–150 nm in diameter, which were secreted by almost all cell types of human body [8]. Therefore, exosomes can be found in a variety of body fluids, such as urine, blood, ascites, and blood. The morphology of exosomes was like a “cup” or a “disc” under a transmission electron microscope, and the expression of specific protein markers, including CD9, CD81, CD63, TSG101, and heat shock protein 70, was used to identify exosomes. By packaging and transferring active molecules, such as proteins, DNAs, mRNAs, and non-coding RNAs from one cell to another, exosomes act as a “postman” to communicate between cells [9]. Such communication also exists between TME cells and tumor cells [10]. Recent studies have shown that BMSC-derived exosomes play a critical role in tumor cell proliferation and migration [11, 12]. A study conducted by Qi et al [13] demonstrated that BMSC-derived exosomes promote osteosarcoma cell growth. However, the mechanism remains unelucidated.

Long non-coding RNAs (lncRNAs), which are a subset of non-coding RNAs with more than 200 bases, have been indicated in regulating tumor growth and metastasis [14]. The lncRNA plasmacytoma variant translocation 1 (PVT1), localized to human band 4 of region 2 on the long arm of chromosome 8 (8q24), is one of the oncogenic lncRNAs. As reported, PVT1 is up-regulated in various malignant tumors, including osteosarcoma [15]. Song et al [16] demonstrated that the upregulation of PVT1 is closely related to the poor prognosis of osteosarcoma patients, and the transfection of PVT1 siRNA markedly inhibits the proliferation of osteosarcoma cells. These findings suggest that PVT1 is critical in the progression of osteosarcoma, while the underlying mechanisms are largely unknown. Since lncRNAs can be packaged in BMSC-derived exosomes and affect tumor growth [11, 17], we assumed that the upregulation of PVT1 in osteosarcoma cells is partly transported by BMSC-derived exosomes, and regulates downstream target expression thus promoting osteosarcoma growth and metastasis.

Herein, the high expression of PVT1 in BMSC-derived exosomes was identified, and the upregulation of PVT1 in osteosarcoma cells was confirmed to be transported from BMSC-derived exosomes. In addition, our findings indicated that PVT1 in BMSC-derived exosomes promotes osteosarcoma cell proliferation and migration via increasing ERG, an oncogenic protein in osteosarcoma, suggesting interfering PVT1 in exosomes as a therapeutic target of osteosarcoma.

## RESULTS

### BMSC-derived exosomes transport lncRNA PVT1 into osteosarcoma cells

**Figure 1:**
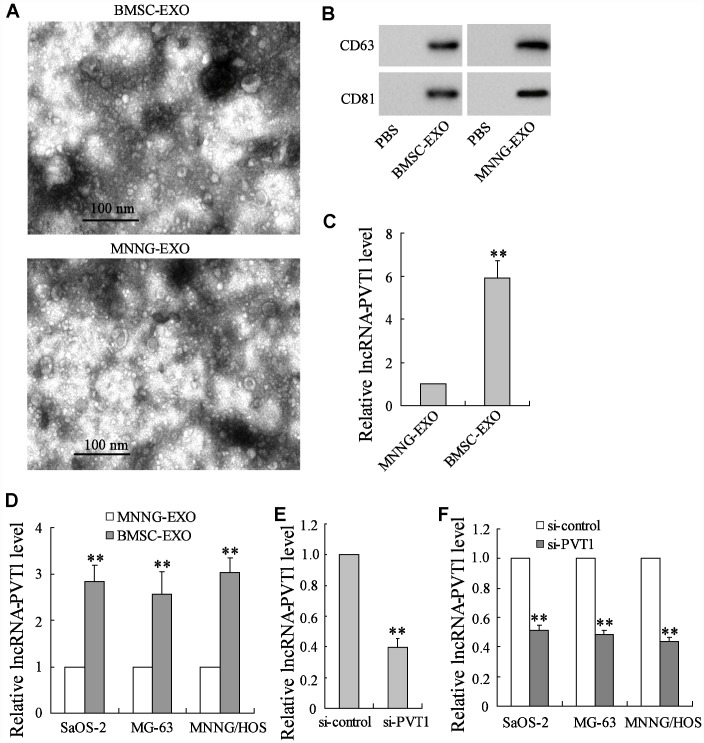
**BMSC-derived exosomes transport lncRNA PVT1 into osteosarcoma cells.** The BMSC-derived exosomes (BMSC-EXO) and the osteosarcoma cell MNNG/HOS-derived exosomes (MNNG-EXO) in the culture medium were respectively isolated. (**A**) They were observed under the transmission electron microscopy. (**B**) The expression of CD81 and CD63 proteins, both of which were exosome markers, was detected using western blot analysis. (**C**) The expression of PVT1 was detected using qRT-PCR. The osteosarcoma cell lines, including Saos-2, MG-63, and MNNG/HOS, were co-cultured with 40 μg/mL BMSC-EXO or MNNG-EXO for 48 h. (**D**) The expression of PVT1 in osteosarcoma cells. (**E**) BMSCs were transfected with si-PVT1 for 48 h. The expression of PVT1 in exosomes which were isolated from PVT1-interfering BMSCs was detected. (**F**) The expression of PVT1 in osteosarcoma cells, which were co-cultured with BMSC-EXO^si-PVT1^ or BMSC-EXO^si-control^ for 48 h. Three independent experiments. **p<0.01 vs MNNG-EXO or si-control. PBS, phosphate buffer saline was used as the control of both kinds of exosomes. si-PVT1, small interfering RNA of PVT1.

The BMSC-derived exosomes (BMSC-EXO) and the osteosarcoma cell MNNG/HOS-derived exosomes (MNNG-EXO) were respectively isolated. They were observed under the transmission electron microscopy, showing typical small round nanoparticles with a diameter from 40 to 150 nm (Figure 1A). Both kinds of exosomes were confirmed by western blot analysis as they expressed CD81 and CD63 proteins which are well-defined exosome markers (Figure 1B). The different expressions of PVT1 were compared between BMSC-EXO and MNNG-EXO using qRT-PCR, and the result showed an elevator of PVT1 expression in BMSC- EXO (Figure 1C), suggesting that the upregulation of PVT1 in osteosarcoma cells may be derived from the transportation of BMSC-EXO. To confirm this hypothesis, the osteosarcoma cell lines, including Saos-2, MG-63, and MNNG/HOS, were co-cultured with BMSC-EXO or MNNG-EXO for 48 h. The co-culture with increasing amounts of BMSC-EXO (from 0 to 40 μg/mL) gradually raised the PVT1 levels in osteosarcoma cells (Supplementary Figure 1A). After the co-culture with 40 μg/mL BMSC-EXO, the PVT1 expression in osteosarcoma cells was markedly increased compared with the co-culture with MNNG-EXO (Figure 1D). Meanwhile, we downregulated the expression of PVT1 in exosomes by interfering PVT1 in BMSCs (Figure 1E), the PVT1 expression in osteosarcoma cells showed a reduction after co-culturing with BMSC-EXO^si-PVT1^ (Figure 1F). In addition, the analysis of exosome concentration (μg exosomes/10^6^ cells) indicated similar quantities of exosomes secreted by PVT1-interfering or control BMSCs (Supplementary Figure 1B), suggesting the interference of PVT1 in BMSCs did not affect the release of exosomes. These data indicated that BMSC-derived exosomes transport PVT1 into osteosarcoma cells and increasing PVT1 expression in osteosarcoma cells.

### PVT1 in BMSC-EXO inhibits degradation and ubiquitination of ERG in osteosarcoma cells

**Figure 2:**
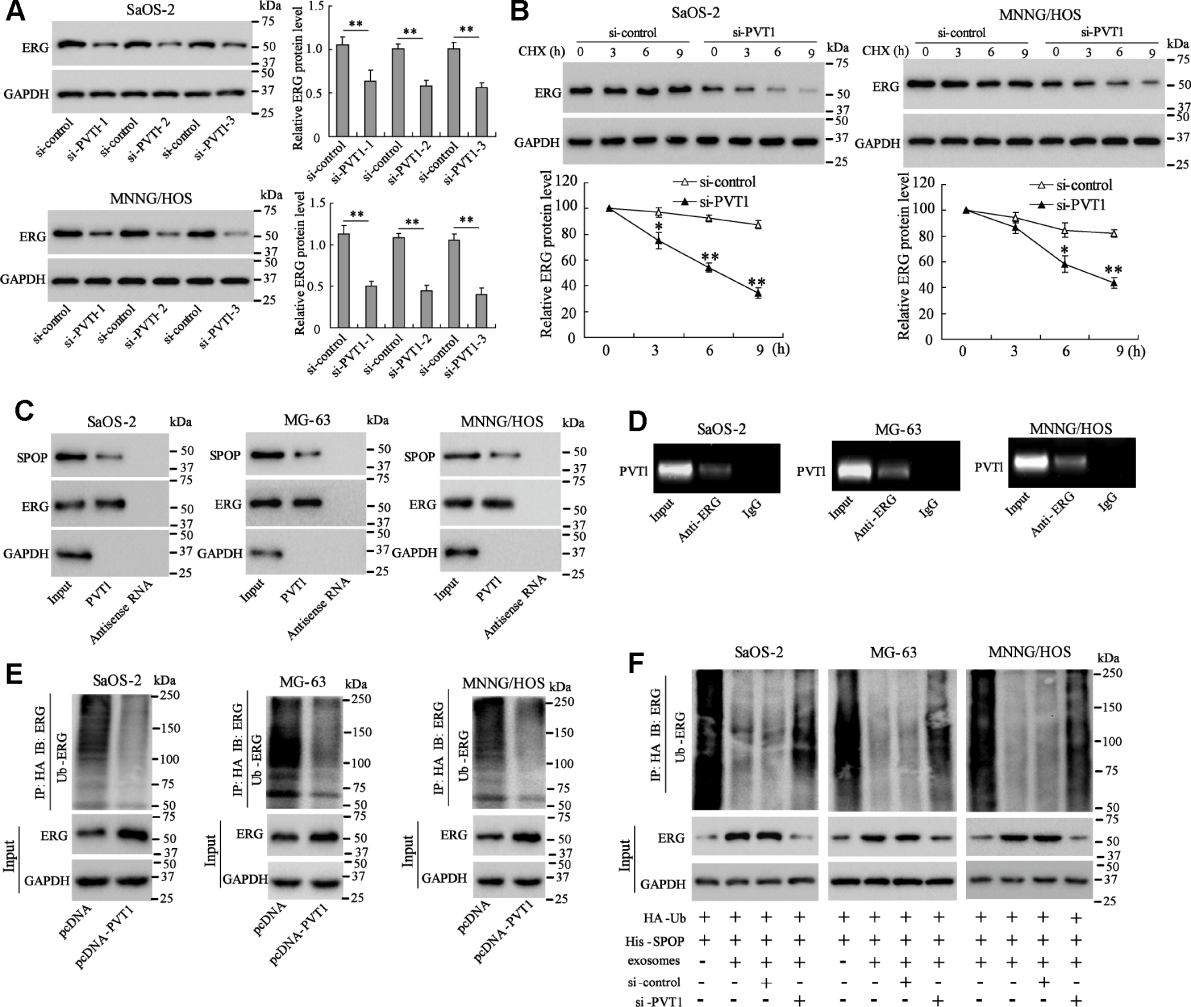
**PVT1 in exosomes inhibits degradation and ubiquitination of ERG in osteosarcoma cells.** Ssos-2 and MNNG/HOS cells were transfected with siRNA of PVT1 (si-PVT1) for 48 h. (**A**) The expression of ERG protein. (**B**) The degradation of ERG protein at 3, 6, and 9 hours after the treatment of the protein synthesis inhibitor, CHX (125 μg/mL). (**C**) The SPOP and ERG proteins were detected in PVT1-protein complex using RNA pull-down assay. Input was used as the positive control; antisense RNA was used as the negative control. (**D**) PVT1 was detected in ERG-RNA binding complex using RIP assay. Input was used as the positive control; IgG was used as the negative control. (**E**) Ubiquitination assay: Ssos-2, MG-63, and MNNG/HOS cells were transfected with pcDNA-PVT1, HA-Ub and His-SPOP for 24 h followed by the immunoprecipitation with HA antibody and immunoblotting with ERG antibody. (**F**) The ubiquitination assay was also performed in PVT1-interfering osteosarcoma cells after being co-cultured with BMSC-EXO. Three independent experiments. *p<0.05, **p<0.01 vs si-control. CHX, cycloheximide. pcDNA-PVT1, the PVT1 overexpressing vector. HA, hemagglutinin. Ub, ubiquitin.

To identify the downstream proteins that interact with PVT1, MG-63 cells were co-cultured with BMSC-EXO and Mass spectrometry was used to detect proteins in PVT1 pull-down complex from co-cultured MG-63 cells. The result showed that the ERG protein expression was increased the most obviously in the PVT1 pull-down complex (Table 1), therefore we further investigated the relationship between PVT1 and ERG. The interfering of PVT1 in Saos-2 and MNNG/HOS cells reduced ERG protein expression (Figure 2A). The ERG protein expression in PVT1-interfering Saos-2 and MNNG/HOS cells was gradually reduced at 3, 6, and 9 hours after the treatment of the protein synthesis inhibitor, suggesting PVT1 is essential for protecting ERG protein from degradation (Figure 2B). These responses were also observed in MG-63 cells (data not shown).

**Table 1:** Proteins found by mass spectrometry analysis in PVT1 pull-down.

**Protein**	**Score**
v-ets avian erythroblastosis virus E26 oncogene homolog (ERG)	45.4
Cellular Glutathione Peroxidase (GPx)	33.6
40S ribosomal protein S16	29.4
Putative beta-actin-like protein 3	22.1
Type 1 cytoskeletal keratin 9	19.9
RNA-binding protein 25	19.9

Ubiquitination plays a critical role in promoting protein degradation. Since the previous study has reported that the degradation and ubiquitination of ERG could be promoted by SPOP, an E3 ubiquitin ligase [18, 19], we further explored whether PVT1 regulates ubiquitination of ERG. The RNA pull-down assay confirmed the binding of SPOP and ERG proteins with PVT1 (Figure 2C), and the RIP assay revealed the existence of PVT1 in ERG-RNA binding complex (Figure 2D). These data suggested the direct interaction between PVT1 and ERG protein. We then overexpressed PVT1 in three osteosarcoma cell lines, and immunoblotted Ub-ERG using ubiquitination assay. The result indicated that overexpressing PVT1 markedly reduced ubiquitin-modified ERG protein (Figure 2E). The ubiquitination assay was also performed in PVT1-interfering osteosarcoma cells after being co-cultured with BMSC-EXO, and the result demonstrated that PVT1 in exosomes inhibited ubiquitination of ERG protein in osteosarcoma cells (Figure 2F).

### PVT1 promotes ERG expression via sponging miR-183-5p

**Figure 3:**
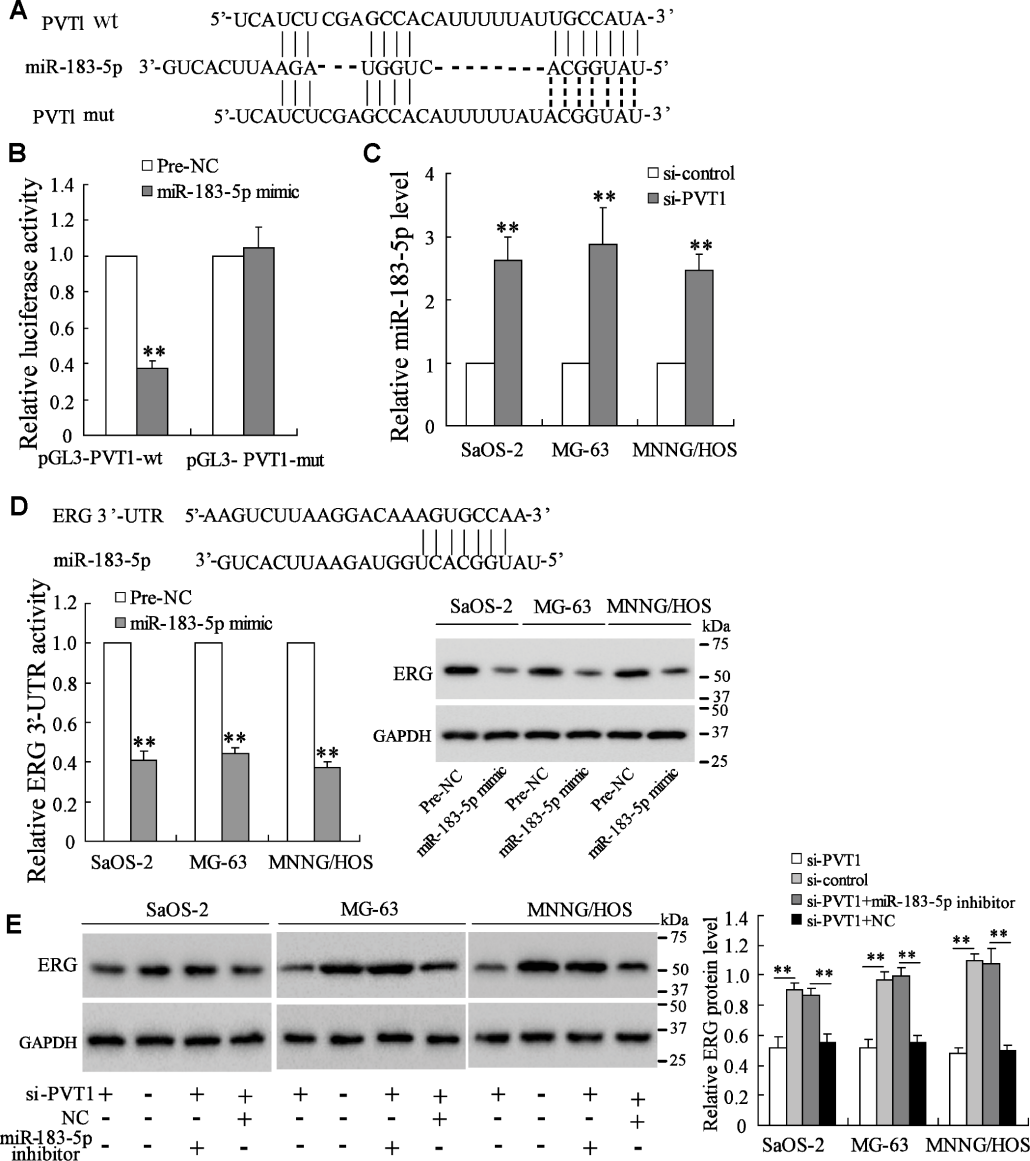
**PVT1 promotes ERG expression via sponging miR-183-5p.** (**A**) The online database predicted the potential binding between PVT1 and miR-183-5p. (**B**) The luciferase activity after the co-transfection of miR-183-5p mimic and pGL3-PVT1-wt or miR-183-5p mimic and pGL3-PVT1-mut. (**C**) The expression of miR-183-5p in PVT1-interfering osteosarcoma cells. (**D**) The online database predicted the interaction between miR-183-5p and ERG 3’-UTR. The luciferase activity of ERG 3’-UTR and the protein level of ERG were detected after overexpressing miR-183-5p. (**E**) The ERG protein expression after the transfection of si-PVT1 or the co-transfection with miR-183-5p inhibitor for 48 h in three osteosarcoma cell lines. Three independent experiments. **p<0.01 vs negative control (pre-NC) or si-control. wt, wild type. mut, mutant. si-PVT1, the siRNA of PVT1. Pre-NC, the control of miR-183-5p mimic. NC, the control of imR-183-5p inhibitor.

As reported, lncRNAs can act as competing endogenous RNAs (ceRNAs) to promote protein expressing by sponging microRNAs [20]. The online database (http://www.targetscan.org/) predicted the potential binding between PVT1 and miR-183-5p (Figure 3A). The luciferase reporter assay showed that the co-transfection with miR-183-5p mimic and pGL3-PVT1-wt vector significantly lowered the luciferase activity, while the co-transfection with miR-183-5p mimic and pGL3-PVT1-mut vector did not affect the luciferase activity (Figure 3B). Meanwhile, the interference of PVT1 in osteosarcoma cells dramatically increased miR-183-5p expression (Figure 3C). These data indicated that miR-183-5p was negatively regulated by PVT1. The online database also predicted the interaction between miR-183-5p and ERG 3’-UTR. The luciferase assay showed that the miR-183-5p mimic reduced the activity of ERG 3’-UTR and the protein expression of ERG (Figure 3D), suggesting that miR-183-5p promoted the down-regulation of ERG by targeting the 3’-UTR of ERG mRNA. Meanwhile, the co-transfection of si-PVT1 and miR-183-5p inhibitor restored the ERG protein expression which was reduced by interfering PVT1 in three osteosarcoma cell lines (Figure 3E).

### PVT1 in BMSC-EXO promotes osteosarcoma cell proliferation and migration via increasing ERG

**Figure 4:**
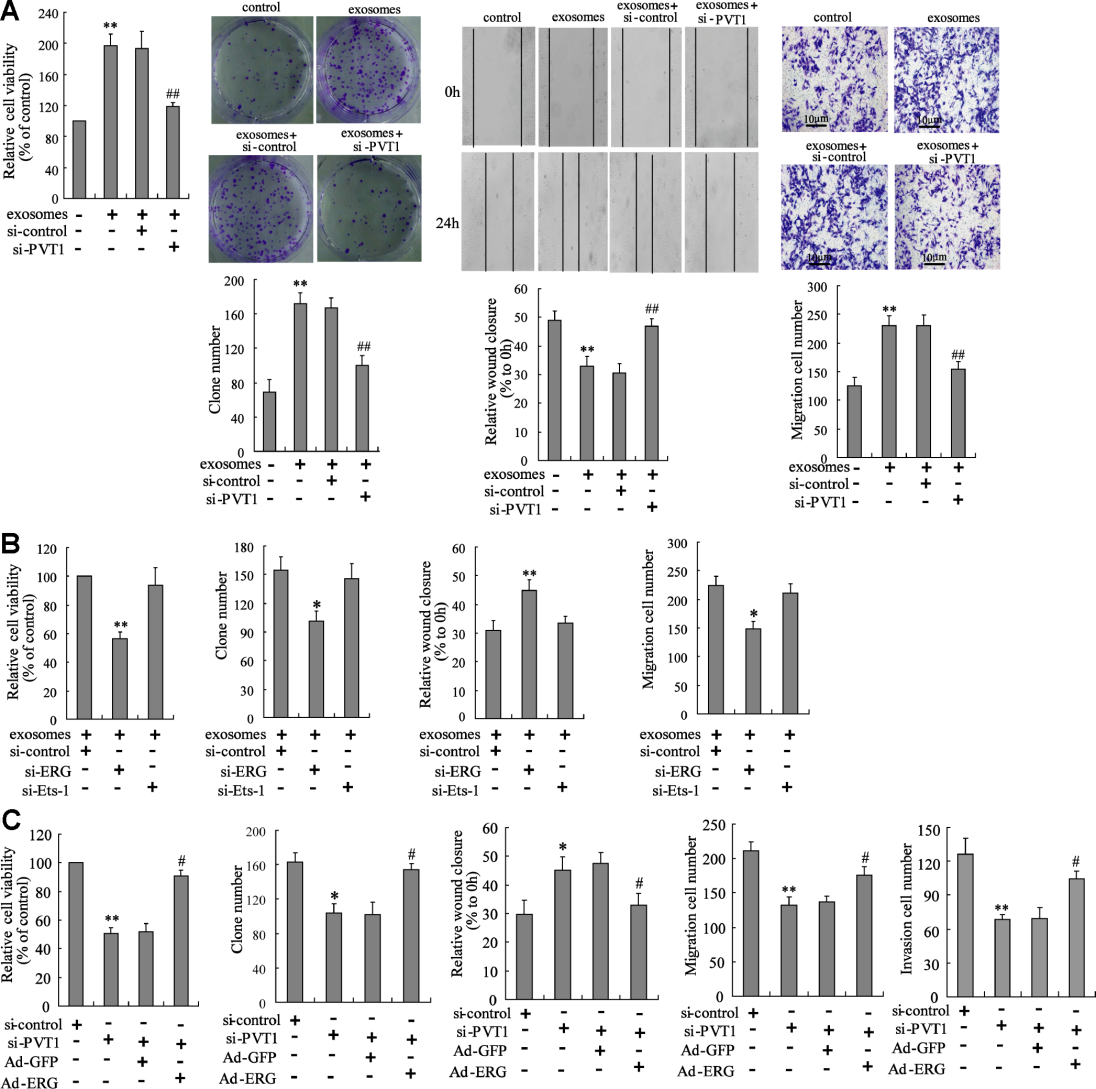
**PVT1 in BMSC-EXO promotes osteosarcoma cell proliferation and migration via increasing ERG.** (**A**) The BMSC-EXO^si-PVT1^ was co-cultured with MNNG/HOS cells for 48 h. After the co-culturing, cell proliferation and migration were detected using CCK-8, colony formation, scratch and Transwell migration assays. (**B**) The MNNG/HOS^si-ERG^ cells and MNNG/HOS^si-Ets-1^ cells were co-cultured with or without BMSC-EXO for 48 h. The cell proliferation and migration were detected. (**C**) The BMSC-EXO^si-PVT1^ was co-cultured with MNNG/HOS cells or MNNG/HOS^Ad-ERG^ cells for 72 h. The cell proliferation, migration, and invasion were detected. Three independent experiments. *p<0.05, **p<0.01 vs negative control or exosomes+si-control or si-control. #p<0.05, ##p<0.01 vs exosomes+si-control or si-PVT1+Ad-GFP. BMSC-EXO^si-PVT1^, exosomes isolated from PVT1-interfering BMSC. MNNG/HOS^si-ERG^ cells, the MNNG/HOS cells which were transfected with siRNA of ERG. MNNG/HOS^si-Ets-1^ cells, the MNNG/HOS cells which were transfected with siRNA of Ets-1. MNNG/HOS^Ad-ERG^, the MNNG/HOS cells which were transfected with ERG-overexpressing vector. CCK-8, cell counting kit-8 assay.

Given that the PVT1 in BMSC-EXO increased ERG protein expression via inhibiting ubiquitination and sponging miR-183-5p, we investigated the mechanism of PVT1 on osteosarcoma cell proliferation and migration. The exosomes isolated from PVT1-interfering BMSC were co-cultured with MNNG/HOS cells. After the co-culturing, cell proliferation and migration were significantly inhibited using CCK-8, colony formation, scratch and Transwell migration assays (Figure 4A). The co-culturing of BMSC-EXO and ERG-interfering MNNG/HOS cells (MNNG/HOS^si-ERG^) reduced the malignancy of osteosarcoma cells, while the co-culturing of BMSC-EXO and Ets-1-interfering MNNG/HOS cells (MNNG/HOS^si-Ets-1^) did not negate such response (Figure 4B). As Ets-1 is a well-defined pro-metastatic factor in osteosarcoma [21], we supposed that the pro-metastatic effect of PVT1 in BMSC-EXO was exerted via specifically raising ERG expression. Moreover, the co-culturing of BMSC-EXO^si-PVT1^ and ERG-overexpressing MNNG/HOS cells (MNNG/HOS^Ad-ERG^) restored the defect of tumor malignancy caused by the co-culturing of BMSC-EXO^si-PVT1^ and MNNG/HOS cells (Figure 4C).

### PVT1 in BMSC-EXO promotes osteosarcoma growth and metastasis via increasing ERG *in vivo*

**Figure 5:**
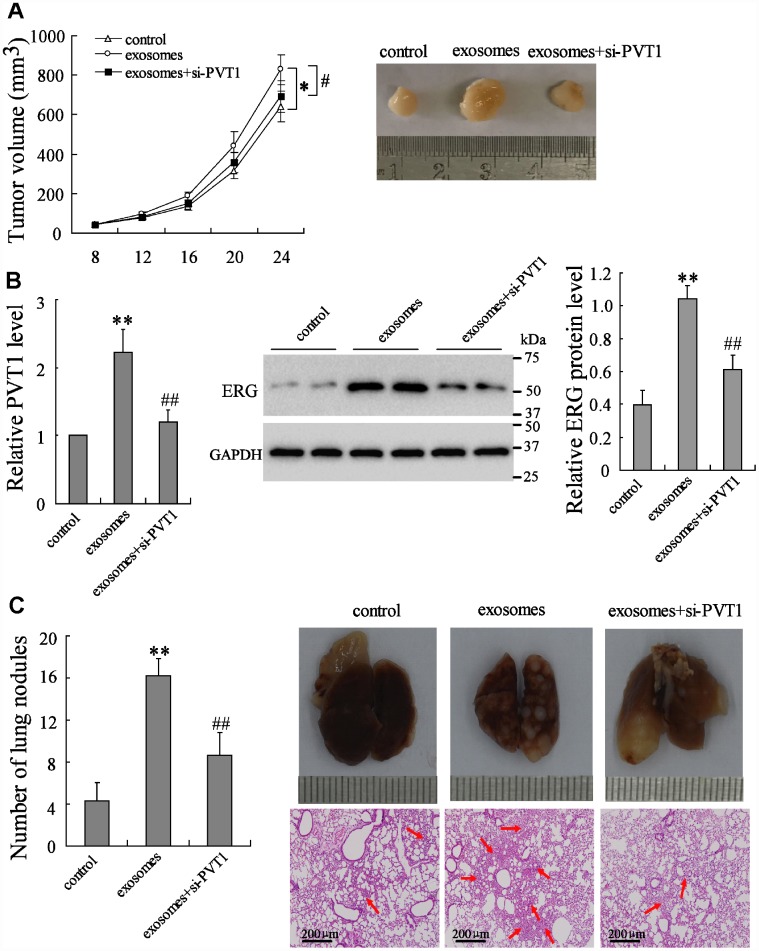
**PVT1 in BMSC-EXO promotes osteosarcoma growth and metastasis via increasing ERG *in vivo.*** The mouse xenograft (n=18) was established by subcutaneous inoculation of MNNG/HOS cells, and the pulmonary metastatic model (n=18) was established by tail vein injection of MNNG/HOS cells. Eight days after the establishment of xenograft and 3 weeks after the establishment of metastatic model, mice were divided into 3 groups, the control group (with PBS injection in tail vein, n=6), the exosome group (with 10 μg of BMSC-EXO injection in tail vein, n=6), and the exosomes+si-PVT1 group (with PVT1-interfering BMSC derived exosome injection in tail vein, n=6). (**A**) The tumor volume was detected every 4 days. (**B**) The expression of PVT1 and ERG in tumor tissues after 3 weeks. (**C**) The number and the H&E staining of lung metastatic nodules (red arrows). *p<0.05, **p<0.01 vs control. #p<0.05, ##p<0.01 vs exosomes.

To elucidate the effect of PVT1/ERG on tumor growth *in vivo*, the mouse xenograft and pulmonary metastatic model were established. Compared with the control group, the BMSC-EXO injection markedly increased tumor growth, while the injection of BMSC-EXO^si-PVT1^ negated such response (Figure 5A). The expression of PVT1 and ERG in tumor tissues was enhanced by BMSC-EXO injection, while it was reduced by BMSC-EXO^si-PVT1^ injection (Figure 5B). In the pulmonary metastatic model, the number of lung metastatic nodules was dramatically increased after BMSC-EXO injection in comparison to the control group, while it was negated by BMSC-EXO^si-PVT1^ injection (Figure 5C). Taken together, these data indicated that PVT1 in BMSC-EXO promotes osteosarcoma growth and metastasis via increasing ERG.

## DISCUSSION

As a major component of the TME, mesenchymal stem cells can be obtained from many kinds of tissues, such as adipose tissue, bone marrow, umbilical cord, and placenta [22]. BMSCs are mesenchymal stem cells isolated from bone marrow, and play an important role in cancer progression. For instance, the direct contact of BMSC with tumor cell inhibits tumor growth in Kaposi’s sarcoma [23]; the combination treatment of TRAIL-expressing BMSCs with doxorubicin promotes breast cancer apoptosis *in vitro* and tumor suppression *in vivo* [24]. These studies indicated the tumor-suppressing effects of BMSCs in TME, while some studies have revealed the tumor-promoting effects of BMSCs. A study conducted by Ho et al [25] suggested that the HDAC3 inhibitor overcomes the anti-apoptotic effect of BMSCs to multiple myeloma cells. In osteosarcoma, Fontanella and his colleagues [26] found that BMSC-conditioned medium promotes osteosarcoma cell (U2OS cell line) growth and migration. Based on these studies, we further investigated the mechanism of tumor-promoting effect of BMSCs to osteosarcoma in the current study, and found that the critical role of BMSC-derived exosomes in regulating tumor cell proliferation and migration.

Exosomes were first reported in 1981, which were extracellular vesicles with 40-150 nm in diameter [9]. The main function of exosomes is to communicate between cells, including between tumor cells and stromal cells in TME, via transporting intracellular components, such as RNAs, DNAs, and proteins [27]. Exosomes can be secreted by various kinds of cell types, including BMSC. Accumulating studies have shown that BMSC-derived exosomes promote or suppress tumor growth through affecting RNA/protein expression of receipt cells, indicating the injection of exogenous exosomes containing active substances as a potential therapeutic strategy. It is reported that the knockdown of HDAC3 in BMSC-derived exosomes results in the decreased multiple myeloma cell proliferation [28], and the delivery of miR-143 by BMSC-derived exosomes suppresses osteosarcoma cell (143B cell line) migration [29]. In our work, we demonstrated that the lncRNA PVT1 is highly expressed in BMSC-derived exosomes, and contributes to the upregulation of PVT1 in osteosarcoma cells (MNNG/HOS, MG-63, and Saos-2 cell lines). Meanwhile, the BMSC-derived exosomes promote osteosarcoma growth and metastasis via PVT1/ERG pathway. After the knockdown of PVT1 in BMSCs, the BMSC-EXO^si-PVT1^ which contains lower amounts of PVT1 than normal BMSC-EXO was obtained, and the effect of BMSC-EXO^si-PVT1^ on osteosarcoma metastasis was inhibited, suggesting that the knockdown of PVT1 in BMSCs exerts the tumor-suppressing effect and may become a novel therapeutic strategy of osteosarcoma. However, whether BMSC-EXO^si-PVT1^ could compete with BMSC-EXO for the uptake by osteosarcoma cells deserves further investigations. Human normal osteoblast cell line (hFOB 1.19) was also co-cultured with increasing amounts of BMSC-EXO (from 0 to 40 μg/mL), and PVT1 expression was raised only by high concentrations of BMSC-EXO (Supplementary Figure 1C). We supposed that exosomes might be selectively taken by osteosarcoma cells rather than normal cells, which has been reported previously [30], and more researches are still needed.

PVT1 is a well-identified oncogenic lncRNA, promoting the progression of numbers of tumor kinds, including non-small-cell lung cancer [31], gallbladder cancer [32], and osteosarcoma [16], and predicting poor overall survival of tumors [33]. There are two main interactions between PVT1 and protein expression. Firstly, PVT1 can act as an ceRNA that sponges miRNAs thus suppressing the inhibition of miRNAs to their targets and promoting target protein expression. As reported, miR-424-5p [31], miR-143 [32], and miR-20a-5p [34] can be sponged by PVT1. Secondly, PVT1 regulates protein expression via direct binding thus affecting the stability of target proteins. The proteins of EZH2 and Lin28 have been reported to directly binding with PVT1, and the stability of Lin28 is affected by PVT1 [35, 36]. In the current study, we demonstrated the two kinds of interactions between PVT1 and ERG. The upregulation of PVT1 suppresses the degradation and ubiquitination of ERG, thus maintaining the stability of ERG. Meanwhile, PVT1 was suggested to promote ERG protein expression via sponging miR-183-5p. These findings revealed the critical role of PVT1 and ERG in the progression of osteosarcoma, adding a new insight into the mechanism of PVT1 and ERG in promoting osteosarcoma growth and metastasis.

ERG, also named as the v-ets avian erythroblastosis virus E26 oncogene homolog, belongs to the highly conserved ETS family [37]. Acting as a transcription factor, ERG is vital for the developmental processes such as bone development, chondrocyte maturation, and hematopoiesis [38]. Shon et al [39] indicated that ERG is highly expressed in osteosarcoma tissues, and suggested ERG as a possible marker of chondroid/ cartilaginous differentiation. In the study conducted by Jumbe et al [40], ERG was demonstrated to increase the bone matrix formation and promote osteosarcoma development via activating the transcription of *TNSALP*. Consistent with their studies, we also observed the upregulation of ERG in osteosarcoma cells and the pro-proliferating and pro-metastatic effect of ERG. In addition, the ERG expression was indicated to be regulated by PVT1 which is transported by BMSC-derived exosomes. However, whether ERG affects the progression of osteosarcoma through other mechanisms still needs more investigations.

In conclusion, our findings indicated that BMSC-derived exosomes promote osteosarcoma growth and metastasis through transporting lncRNA PVT1 to osteosarcoma cells. Furthermore, the upregulation of PVT1 resulted in the stabilization of ERG protein by inhibiting ubiquitination of ERG, and also resulting in the upregulation of ERG expression by competely binding with miR-183-5p. This study provided a new insight into the mechanism of BMSC-derived exosomes in modulating osteosarcoma progression.

## MATERIALS AND METHODS

### Clinical samples

Human BMSCs were isolated from bone marrows which were collected from middle-aged patients with femoral fractures or candidates for artificial hip joint replacement. All the patients were absent of blood diseases. During the surgery, a total of 5–6 ml fresh bone marrows was obtained after the removal of fracture ends or the femoral head followed by the expansion of the medullary cavity. After the incubation of 4–7 days, the growth of adherent mesenchymal stem cells reached 80–90%, and the passage was carried out. The isolation of exosomes was performed in the 3^rd^ to 6^th^ generation of BMSCs.

### Cell line and cell culture

Human osteosarcoma cell lines (MNNG/HOS, Saos-2, and MG-63) were purchased from American Tissue Culture Collection (ATCC). MNNG/HOS cells (CRL-1547™) were cultured in the ATCC-formulated Eagle's Minimum Essential Medium (EMEM, 30-2003^TM^) supplemented with 1% penicillin-streptomycin and 10% exosome-depleted fetal bovine serum (FBS, ATCC, 30-2020^TM^) in which the exosomes were depleted by ultracentrifugation. Saos-2 cells (HTB-85^TM^) were cultured in the ATCC-formulated McCoyʼs 5a Medium Modified (30-2007 ^TM^). MG-63 cells were cultured in the ATCC-formulated EMEM (30-2003^TM^). All the cells were cultured in an incubator with a humid atmosphere of 5% CO_2_ at 37 °C.

### Exosome isolation

The BMSC and MNNG/HOS cell culture supernatants which contain exosomes were harvested. The purification of the exosomes was performed by ultracentrifugation using a Ti70 rotor (optima L-100 XP Ultracentrifuge, Beckman Coulter, Brea, CA, USA) as previously described [13]. Briefly, the harvested supernatants were centrifuged at 300 ×g for 10 min followed by 2000 ×g for 10 min to remove cells. Then, the supernatants were centrifuged at 10000 ×g for 30 min in order to remove cell debris. The supernatants were collected and ultracentrifugated at 100000 ×g for 70 min, twice. After discarding the supernatants, we obtained the pellets containing exosomes. All the centrifugations were performed at 4°C and the aliquots were filtered using 0.22-μm filters. The pellets were resuspended in 200 μl phosphate buffer saline (PBS) for use or stored at −80 °C. The isolated exosomes were named as BMSC-EXO from BMSCs and MNNG-EXO from MNNG/HOS cells, respectively.

### Exosome identification

A total of 10 μL PBS solution was added into an equal volume of isolated exosomes to identify the exosomes. The sample was added dropwise to a 2 mm copper mesh. After keeping at room temperature for 1 min, the excess liquid was gently removed followed by the staining using 3% (w/v) sodium phosphotungstate solution for 1 min at room temperature. Then the sample was gently washed and air-dried at room temperature. The shape of exosomes was observed under a transmission electron microscope (HT7830, HITACHI, Ltd., Tokyo, Japan). Western blot analysis was used to detect the expression of exosome markers CD63 and CD81. Exosome quantification was determined by the Bradford method (Bio-Rad Laboratories, Hercules, CA, USA).

### Western blot analysis

The RIPA lysate containing phenylmethanesulfonyl fluoride (PMSF, Beyotime, Beijing, China), a protease inhibitor, was added into the exosomes or treated cells, and lysed on cold ice for 30 min. Then they were centrifuged and the supernatant containing proteins was collected for SDS-PAGE electrophoresis. After protein quantification by BCA kit (Thermo Fisher Scientific, Waltham, MA, USA), each sample of equal mass was subjected to 10% separation gel SDS-PAGE electrophoresis. The separated protein was then transferred to the PVDF membrane, blocked for 2 h (5% skim milk powder prepared by TBST). They were incubated with the primary antibodies, including anti-CD63 (1:000, ab217345, Abcam, Cambridge, UK), anti-CD81 (ab79559, Abcam), anti-ERG (1:1000, ab92513, Abcam), anti-SPOP (1:500, ab137537, Abcam), anti-β-actin (1:500, ab8226, Abcam), anti-GAPDH (1:500, ab8245, Abcam), at 4 °C overnight. The next day, the membrane was incubated with horseradish peroxidase-labeled secondary antibody (1:2000, ab205718, Abcam) for 2 h at room temperature. An enhanced chemiluminescence kit (Pierce Chemical Co., Rockford, IL, USA) was used to visualize the protein bands on the transfer membranes.

### Quantitative real-time PCR (qRT-PCR)

The RNA expression in cells or exosomes were detected using qRT-PCR. In brief, cells were lysed using Trizol (Thermo Fisher Scientific, Waltham, MA, USA) and total RNAs in cells or exosomes were extracted by chloroform-isoamyl alcohol method. After qualitatively and quantitatively extracting the sample RNAs, the reverse transcription reaction is carried out according to the description of the TAKARA reverse transcription kit (Promega, Madison, WI, USA). The cDNA is used for the fluorescence quantitative PCR reaction. The qRT-PCR was performed by using Power SYBR Green PCR Master Mix (Thermo Fisher Scientific, Waltham, MA, USA) on a PRISM 7500 Real-TIME PCR System (Applied Biosystems, Waltham, MA, USA). GAPDH was used as the internal control in measuring PVT1 expression in cells and tissues. U6 was used as the internal control in measuring miR-183-5p expression. When measuring exosomal PVT1 expression, a synthesized exogenous control, 0.1 ng (1.8×10^8^ copies) λ polyA^+^ RNA (Takara), was spiked in to normalize the exosomal total RNA at the onset of RNA reverse transcription [41]. The relative value of RNA expression was calculated by the 2^-ΔΔCT^ method.

### PVT1-interfering exosome

BMSCs were transiently transfected with the small interfering RNA of PVT1, named as si-PVT1, using Lipofectamine^TM^ 2000 reagent (Invitrogen, Waltham, MA, USA) for 48 hours according to the manufacturer’s instructions. The si-PVT1 and its control (si-control) were bought from GeneChem (Shanghai, China). The PVT1-interfering exosome was isolated from the PVT1-interfering BMSCs.

### Cell transfection

The osteosarcoma cells (Saos-2, MG-63, and MNNG/ HOS) were transiently transfected with PVT1 overexpressing vector (pcDNA-PVT1), siRNA of ERG (si-ERG), siRNA of Ets-1 (si-Ets-1), ERG overexpressing adenovirus vector (Ad-ERG), or miR-183-5p inhibitor using Lipofectamine^TM^ 2000 reagent (Invitrogen, Waltham, MA, USA). The pcDNA-PVT1 and its control (pcDNA), the si-ERG and its control (si-control), Ad-ERG and its control (Ad-GFP), and miR-183-5p and its control (negative control, NC) were bought from GeneChem (Shanghai, China).

### Cycloheximide (CHX) experiment

The osteosarcoma cells (Saos-2, MG-63, and MNNG/HOS) were transiently transfected with siRNA of PVT1 (si-PVT1) followed by the treatment of a protein synthesis inhibitor, CHX (125 μg/mL). The protein level of ERG was detected at 0, 3, 6, and 9 hours using western blot analysis, respectively.

### RNA pull-down assay

A total of 3 μg biotin-labeled PVT1 was supplemented to 100 μL with RNA structure buffer followed by the denature at 90 °C for 5 min before being placed on ice for 2 min. Then they were transferred to room temperature for 20 min. Streptavidin beads washed with 60 μl of cell lysate were pre-incubated for 1 h at room temperature. Then the biotin-labeled PVT1 and cell lysate were incubated at room temperature for 1 h. The 60 μL of streptavidin beads which were washed with cell lysate were added in the incubation mixture, incubating for 1 h at room temperature. The beads were centrifuged at 3000 rpm for 1 min at 4 °C. After discarding the supernatant, the beads were washed twice with 1 ml of low-salt lotion for 10 min, then washed twice with high-salt lotion for 10 min. The expression of SPOP and ERG in the complex was detected using western blot analysis.

### RNA immunoprecipitation (RIP)

The osteosarcoma cells (Saos-2, MG-63, and MNNG/HOS) were washed twice with PBS. After adding the cell lysis buffer (containing protease inhibitor), the cells were lysed on ice for 30 min. Then they were centrifuged at 14000 g for 15 min at 4 °C. The antibody against ERG was added (the control group was added with normal mouse IgG), and the mixture was incubated at 4 °C overnight. The pre-treated agarose beads of protein A were added to the mixture and incubated at 4 °C for 2-4 h to couple the antibody to the Protein A. After immunoprecipitation, centrifugation was carried out for 3 min at 4 °C, and the supernatant was discarded. The beads were washed 3–4 times with lysis buffer. Then the SDS loading buffer was added, and the expression of PVT1 in the RNA-protein complex was detected by reverse transcriptional PCR and agarose gel electrophoresis.

### Ubiquitination assay

The osteosarcoma cells (Saos-2, MG-63, and MNNG/ HOS) were transfected with hemagglutinin-labeled ubiquitin (HA-Ub), His-labeled SPOP (His-SPOP), and pcDNA-PVT1 for 48 h, and treated with MG132, a protease inhibitor, for 2 hours. Immunoprecipitation was performed using the anti-HA antibody, the immunoprecipitants were separated by SDS-PAGE, and immunoblotted with the anti-ERG antibody to detect the ubiquitination of ERG.

The BMSC derived exosomes were co-cultured with the osteosarcoma cells (Saos-2, MG-63, and MNNG/HOS) which were transfected with HA-Ub, His-SPOP, and si-PVT1. Immunoprecipitation was performed using the anti-HA antibody, and immunoblotting was performed using the anti-ERG antibody.

### Luciferase reporter assay

The PVT1 3'UTR sequence was cloned into the recombinant expression vector to obtain the PVT1 3'UTR target fragment, which was ligated into the dual luciferase reporter vector pGL3-Basic to obtain the wild type reporter vector (pGL3-PVT1-wt). According to the binding site of miR-183-5p in the 3'UTR sequence of PVT1, the mutant PVT1 3'UTR sequence was designed and synthesized, and the mutant reporter vector (pGL3-PVT1-mut) was obtained. The miR-26a-5p mimic and pGL3-PVT1-wt (or pGL3-PVT1-mut) were transfected into 293T cells using the lipofectamine 2000 transfection kit. Cells were harvested 72 h after transfection. The relative fluorescence activity was detected by the luciferase assay.

### Cell counting kit-8 (CCK-8) assay

Cells from each group were seeded in a 96-well plate. A total of 100 μL CCK-8 reagent was added to each well. After mixing the wells, we placed them in an incubator, and incubated them for 4 h. Then the 96-well plate was removed from the incubator and placed on a shaker for 1 min. The absorbance at 450 nm was measured with a microplate reader, and cell viability was calculated based on the absorbance value.

### Colony formation assay

The exosomes isolated from PVT1-interfering BMSCs were co-cultured with MNNG/HOS cells. The cells at a density of 10^3^ cells/mL were inoculated in a 60 mm culture dish. When the cell colonies were visible in the dish, the medium was discarded, and the cells were washed with PBS, fixed with 4% paraformaldehyde, and stained with 0.1% crystal violet. The colonies were observed and photographed.

### Scratch migration assay

The scratch migration assay was performed as described before [42]. The exosomes isolated from PVT1-interfering BMSCs were co-cultured with MNNG/HOS cells in the logarithmic growth phase. Then the cells were seeded in a 12-well plate, and a sterile 10 μl plastic micropipette tip was used to scratch through the monolayer. Then the cells were washed with physiological saline for 3 times, and replaced with DMEM medium containing 0.2% FBS. After making the scratches for 24 h, the migration of the cells was observed using a microscope.

### Transwell migration and invasion assay

The exosomes isolated from PVT1-interfering BMSCs were co-cultured with MNNG/HOS cells. For the migration assay, the above cells were collected and adjusted to a cell density of 10^5^ cells/mL. Then the cells were seeded into the upper chamber. The conventional medium containing FBS was added to the lower chamber. The cells were cultured in an incubator at 37 °C with 1% O_2_, and 5% CO_2_ for 24 h. The cells in the lower chamber were fixed with 4% paraformaldehyde, stained with 1% crystal violet, and observed under a microscope. Five 400-fold fields of view were randomly selected and the number of migrating cells was calculated. For the invasion assay, the experiments were the same as the migration assay, except that the upper chamber was coated with Matrigel (Sigma, St.Louis, MO, USA).

### Xenograft model and pulmonary metastatic model

The immunodeficient BALB/c female nude mice aging from 6 to 8 weeks were bought from the Experimental Animal Center of Zhengzhou University. MNNG/HOS cells at a density of 5 × 10^6^ cells/200 μL PBS were subcutaneously inoculated into the back of the mice. After the injection of 8 days, mice were randomly divided into 3 groups, the control group (with PBS injection in tail vein, n=6), the exosome group (with 10 μg of BMSC derived exosome injection in tail vein, n=6), and the exosomes+si-PVT1 group (with PVT1-interfering BMSC derived exosome injection in tail vein, n=6). The tumor size was measured every 3 days using vernier caliper. For establishing pulmonary metastatic model, another 18 mice were i.v. injected with MNNG/HOS cells before being divided into 3 groups. After 3 weeks, the total of 36 mice was sacrificed, and tumor tissues of the xenograft model and lungs of the metastatic model were harvested for further experiments.

### Statistical analysis

Data were presented as mean ± standard deviation (SD). SPSS software (version 20.0; Chicago, USA) was used in analysis. The significance of difference was detected using t test or one-way analysis of variance (ANOVA). We consider A p value less than 0.05 as statistically significant.

### Ethics approval

This study was approved by the Institute Research Medical Ethics Committee of The First Affiliated Hospital of Zhengzhou University. Written informed consents were obtained from all the patients involved. All animal experiments were approved by the Institute Research Medical Ethics Committee of The First Affiliated Hospital of Zhengzhou University, and all animals-treatment operations were executed according to the Zhengzhou University Ethical Guidelines for Animal Experiment.

## Supplementary Material

Supplementary Figure 1
