# Correction for: Long non-coding RNA PVT1 encapsulated in bone marrow mesenchymal stem cell-derived exosomes promotes osteosarcoma growth and metastasis by stabilizing ERG and sponging miR-183-5p

**DOI:** 10.18632/aging.203990

**Published:** 2022-03-30

**Authors:** Wei Zhao, Pan Qin, Da Zhang, Xichun Cui, Jing Gao, Zhenzhu Yu, Yuting Chai, Jiaxiang Wang, Juan Li

**Affiliations:** 1 Department of Pediatric Surgery, The First Affiliated Hospital of Zhengzhou University, Zhengzhou, 450052, China; 2 Department of Respiratory Medicine, The First Affiliated Hospital of Zhengzhou University, Zhengzhou, 450052, China

Original article: Aging. 2019; 11:9581–9596. https://doi.org/10.18632/aging.102406

**This article has been corrected:** The authors requested replacement of **Figures 2** and **4**, where they found errors in panels 2D and 4A. The image of the "MG-63" group in **Figure 2D** was wrongly obtained from the "MNNG/HOS" group and has been replaced with the correct image from the initial set of experiments. While combining images from the colony formation assays for panel 4A, the colony formation image for the “control” group was mixed up with the "exosomes + si-PVT1" group and has now replaced with a “control” group image from the original set of experiments. These corrections do not change the content of the publication and do not affect the conclusion drawn from this research. The authors would like to apologize for any inconvenience caused.

New **Figures 2** and **4** are presented below.

**Figure 2:**
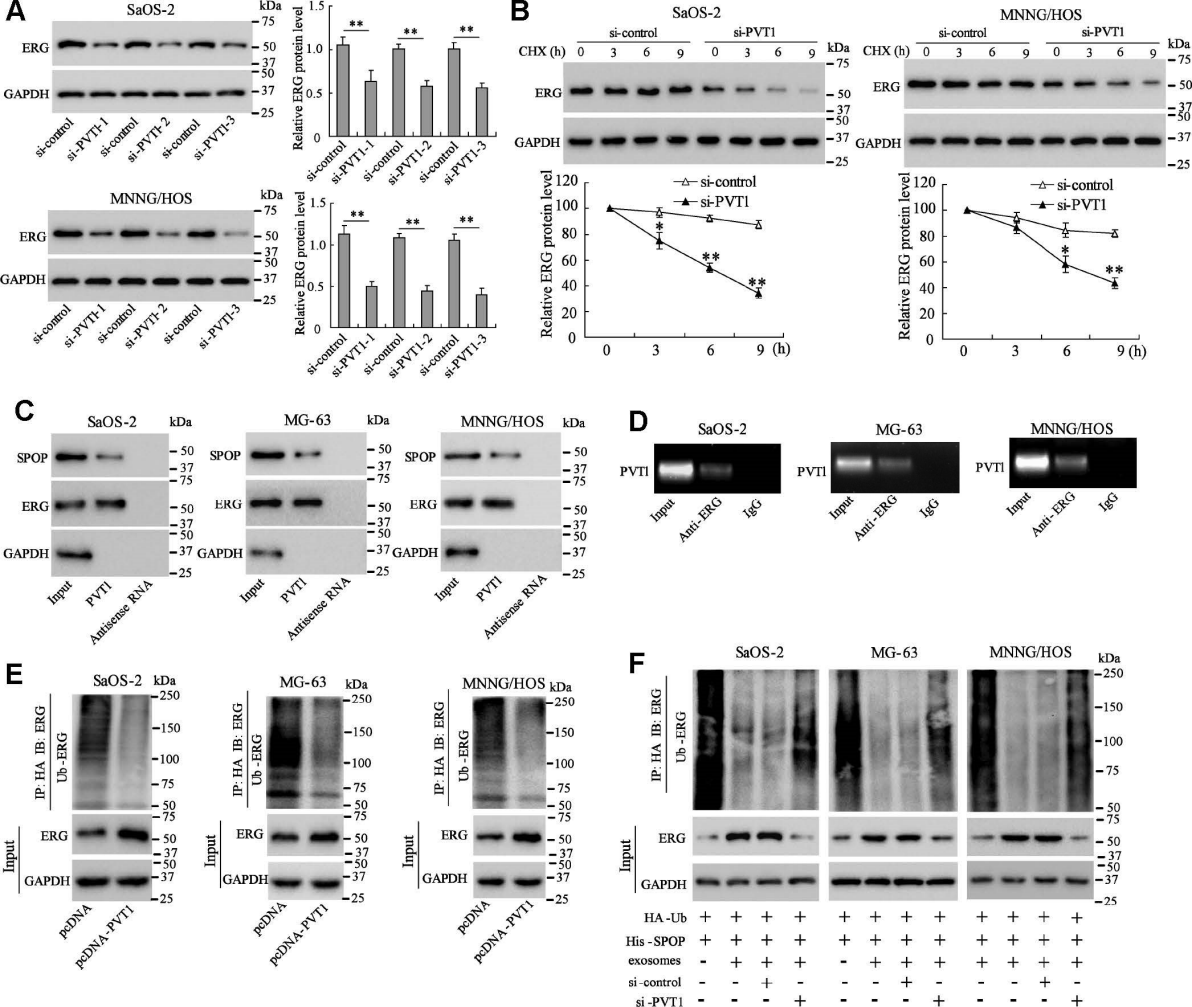
**PVT1 in exosomes inhibits degradation and ubiquitination of ERG in osteosarcoma cells. **Ssos-2 and MNNG/HOS cells were transfected with siRNA of PVT1 (si-PVT1) for 48 h. (**A**) The expression of ERG protein. (**B**) The degradation of ERG protein at 3, 6, and 9 hours after the treatment of the protein synthesis inhibitor, CHX (125 μg/mL). (**C**) The SPOP and ERG proteins were detected in PVT1-protein complex using RNA pull-down assay. Input was used as the positive control; antisense RNA was used as the negative control. (**D**) PVT1 was detected in ERG-RNA binding complex using RIP assay. Input was used as the positive control; IgG was used as the negative control.  (**E**) Ubiquitination assay: Ssos-2, MG-63, and MNNG/HOS cells were transfected with pcDNA-PVT1, HA-Ub and His-SPOP for 24 h followed by the immunoprecipitation with HA antibody and immunoblotting with ERG antibody. (**F**) The ubiquitination assay was also performed in PVT1-interfering osteosarcoma cells after being co-cultured with BMSC-EXO. Three independent experiments. *p<0.05, **p<0.01 vs si-control. CHX, cycloheximide. pcDNA-PVT1, the PVT1 overexpressing vector. HA, hemagglutinin. Ub, ubiquitin.

**Figure 4:**
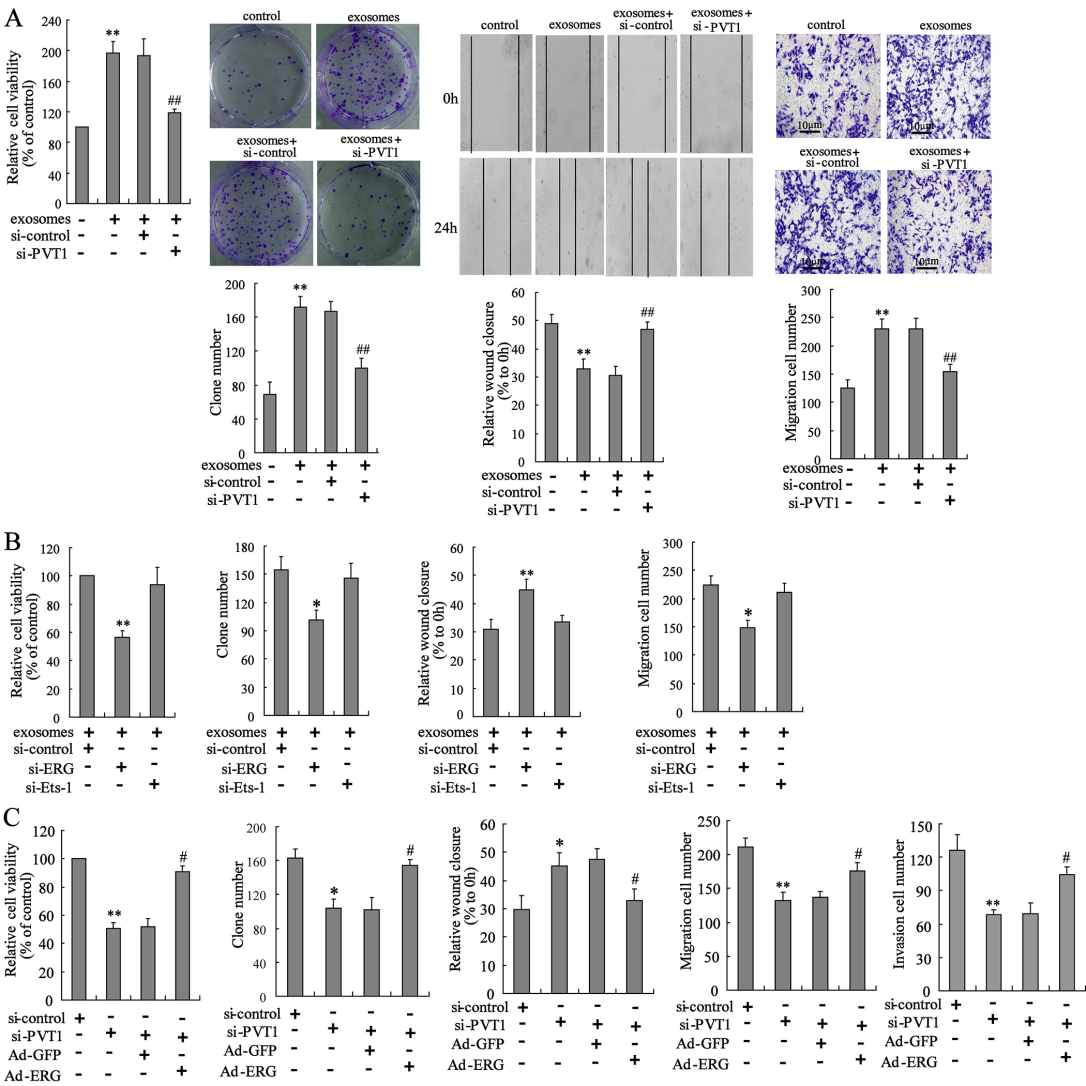
**PVT1 in BMSC-EXO promotes osteosarcoma cell proliferation and migration via increasing ERG. **(**A**) The BMSC-EXO^si-PVT1^ was co-cultured with MNNG/HOS cells for 48 h. After the co-culturing, cell proliferation and migration were detected using CCK-8, colony formation, scratch and Transwell migration assays. (**B**) The MNNG/HOS^si-ERG^ cells and MNNG/HOS^si-Ets-1^ cells were co-cultured with or without BMSC-EXO for 48 h. The cell proliferation and migration were detected. (**C**) The BMSC-EXO^si-PVT1^ was co-cultured with MNNG/HOS cells or MNNG/HOS^Ad-ERG^ cells for 72 h. The cell proliferation, migration, and invasion were detected. Three independent experiments. *p<0.05, **p<0.01 vs negative control or exosomes+si-control or si-control. #p<0.05, ##p<0.01 vs exosomes+si-control or si-PVT1+Ad-GFP. BMSC-EXO^si-PVT1^, exosomes isolated from PVT1-interfering BMSC. MNNG/HOS^si-ERG^ cells, the MNNG/HOS cells which were transfected with siRNA of ERG. MNNG/HOS^si-Ets-1^ cells, the MNNG/HOS cells which were transfected with siRNA of Ets-1. MNNG/HOS^Ad-ERG^, the MNNG/HOS cells which were transfected with ERG-overexpressing vector. CCK-8, cell counting kit-8 assay.

