# Supplementary material for: Long non-coding RNA PVT1 encapsulated in bone marrow mesenchymal stem cell-derived exosomes promotes osteosarcoma growth and metastasis by stabilizing ERG and sponging miR-183-5p
Source: Aging (Albany NY). 2019 Nov 7;11(21):9581–96. doi: 10.18632/aging.102406 (PMC6874467; doi:10.18632/aging.102406)
Supplement: Supplementary Figure 1 [file aging-11-102406-s001.pdf]

SUPPLEMENTARY FIGURE

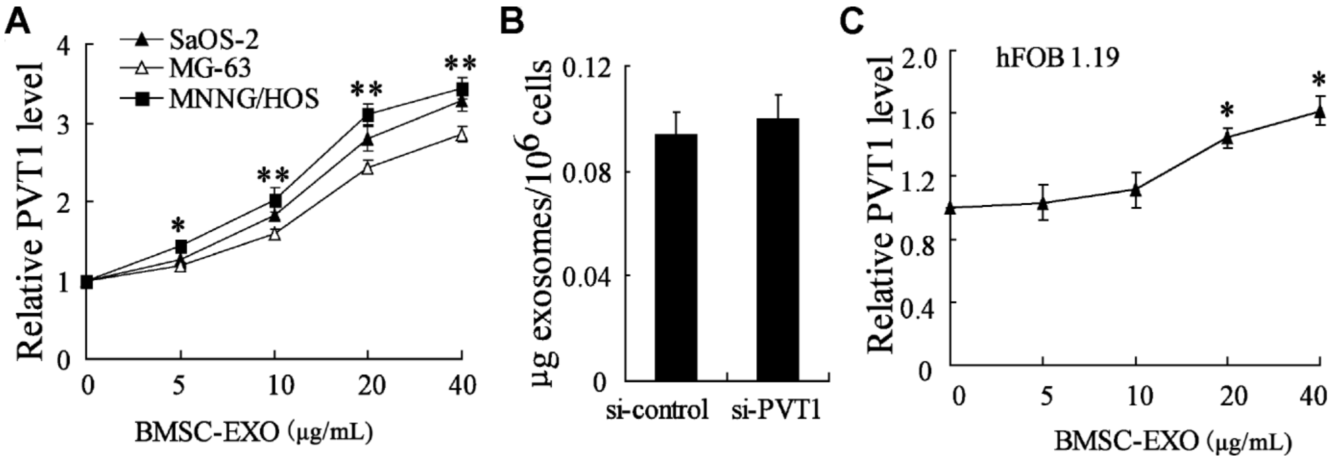

Supplementary Figure 1. (**A**) The expression of PVT1 in osteosarcoma cells after the co-culture with increasing amounts of BMSC-EXO (from 0 to 40 μg/mL) for 48 h. (**B**) The concentration of exosomes which were isolated from PVT1-interfering BMSCs (si-PVT1) or control BMSCs (si-control). si-PVT1, small interfering RNA against PVT1. (**C**) The expression of PVT1 in human normal osteoblast cell line (hFOB 1.19) after the co-culture with increasing amounts of BMSC-EXO (from 0 to 40 μg/mL) for 48 h. *p<0.05, **p<0.01 vs 0 μg/mL BMSC-EXO.
